# SMIXnorm: Fast and Accurate RNA-Seq Data Normalization for Formalin-Fixed Paraffin-Embedded Samples

**DOI:** 10.3389/fgene.2021.650795

**Published:** 2021-03-24

**Authors:** Shen Yin, Xiaowei Zhan, Bo Yao, Guanghua Xiao, Xinlei Wang, Yang Xie

**Affiliations:** ^1^Department of Population and Data Sciences, Quantitative Biomedical Research Center, The University of Texas Southwestern Medical Center, Dallas, TX, United States; ^2^Department of Statistical Science, Southern Methodist University, Dallas, TX, United States

**Keywords:** RNA-sequencing, normalization, FFPE, formalin-fixed paraffin-embedded samples, archived samples, statistical methods

## Abstract

RNA-sequencing (RNA-seq) provides a comprehensive quantification of transcriptomic activities in biological samples. Formalin-Fixed Paraffin-Embedded (FFPE) samples are collected as part of routine clinical procedure, and are the most widely available biological sample format in medical research and patient care. Normalization is an essential step in RNA-seq data analysis. A number of normalization methods, though developed for RNA-seq data from fresh frozen (FF) samples, can be used with FFPE samples as well. The only extant normalization method specifically designed for FFPE RNA-seq data, MIXnorm, which has been shown to outperform the normalization methods, but at the cost of a complex mixture model and a high computational burden. It is therefore important to adapt MIXnorm for simplicity and computational efficiency while maintaining superior performance. Furthermore, it is critical to develop an integrated tool that performs commonly used normalization methods for both FF and FFPE RNA-seq data. We developed a new normalization method for FFPE RNA-seq data, named SMIXnorm, based on a simplified two-component mixture model compared to MIXnorm to facilitate computation. The expression levels of expressed genes are modeled by normal distributions without truncation, and those of non-expressed genes are modeled by zero-inflated Poisson distributions. The maximum likelihood estimates of the model parameters are obtained by a nested Expectation-Maximization algorithm with a less complicated latent variable structure, and closed-form updates are available within each iteration. Real data applications and simulation studies show that SMIXnorm greatly reduces computing time compared to MIXnorm, without sacrificing the performance. More importantly, we developed a web-based tool, *RNA-seq Normalization (RSeqNorm)*, that offers a simple workflow to compute normalized RNA-seq data for both FFPE and FF samples. It includes SMIXnorm and MIXnorm for FFPE RNA-seq data, together with five commonly used normalization methods for FF RNA-seq data. Users can easily upload a raw RNA-seq count matrix and select one of the seven normalization methods to produce a downloadable normalized expression matrix for any downstream analysis. The R package is available at https://github.com/S-YIN/RSEQNORM. The web-based tool, *RSeqNorm* is available at http://lce.biohpc.swmed.edu/rseqnorm with no restriction to use or redistribute.

## 1. Introduction

The application of next-generation sequencing (NGS) on measuring transcript abundance is widely known as RNA-seq. RNA-seq works by sequencing a library of cDNA fragments in a high-throughput manner in order to provide a comprehensive quantification of transcriptomic activities in biological samples (Quinn et al., 2018). In practice, normalization is an important step in RNA-seq data analysis since raw counts are often not directly comparable between samples (Dillies et al., 2013). Normalization brings out the biologically relevant information in gene expression by removing the systematic noise that arises from various experimental reasons (such as batch effect, lane effect, sequencing bias, etc.). Recent studies have shown that the raw sequencing data without normalization could cause invalid inference from many conventional statistical analyses and measurements (Quinn et al., 2018).

Fresh-frozen (FF) tissue biospecimens are considered the gold standard in molecular analysis using gene expression, as freezing preserves RNA well. A number of normalization methods have been well-studied on FF bulk RNA-seq data (Dillies et al., 2013). Most existing methods, including Reads Per Million (RPM), Upper-Quartile (UQ), DESeq, Trimmed Mean of *M*-values (TMM), etc., are based on scaling factor estimation, where the normalized expression is obtained by dividing the raw count by an estimate of the sample-specific scaling factor. For example, RPM (Mortazavi et al., 2008) estimates the scaling factor for each sample by the total number of reads divided by 1, 000, 000. Similarly, UQ (Bullard et al., 2010) estimates the scaling factor by the upper quartile of counts across all genes for each sample. DESeq (Anders and Huber, 2010) normalization first calculates the geometric means of the counts for all genes as an average reference library. Then the ratio of the count in each sample to that in the reference library is computed. The scaling factor for each sample is estimated as the median of this ratio across all genes. TMM (Robinson and Oshlack, 2010) normalization selects one sample as a reference, and the *M*-values are calculated as the log ratios of the read count between each test sample and the reference for all genes. Then for each test sample, the scaling factor is estimated by the weighted mean of *M*-values after removing the genes with extreme average expression or *M*-values. On the other hand, normalization can be done implicitly by accounting for the size factor as a term in the RNA-seq data model. PoissonSeq (PS) (Li et al., 2012) models RNA-seq data by a Poisson log-linear model, where a set of sample-specific parameters is included as offset parameters in the linear predictor to account for different sequencing depths.

Recently, there has been increasing interest in performing transcriptome profiling on Formalin-Fixed Paraffin-Embedded (FFPE) tissues, as they are widely available from routine diagnostic sample preparation (Morton et al., 2014). Being able to successfully measure mRNA abundance from FFPE samples could greatly facilitate biomarker discoveries and genomic studies of clinical samples (Graw et al., 2015; Grenier et al., 2017). The major challenge of adapting FFPE biospecimens in molecular analysis is that the chemical process is designed to preserve cellular proteins rather than preserving RNA. As a result, RNA from FFPE tissues is usually degraded, which could limit gene expression analysis. Studies have shown that the fixation process, storage time, specimen size and conditions play important roles in the RNA quality from FFPE samples (Von Ahlfen et al., 2007). Although such samples may suffer from the chemical modifications and continued degradation over time, recent studies have shown that RNA-seq can measure the RNA expression from FFPE samples in sufficient quality (Li et al., 2014). Due to chemical modifications and the RNA degradation, the RNA quality and abundance extracted from the FFPE samples vary a lot. Therefore, the normalization step is even more important for RNA-seq data measured from FFPE samples than those measured from FF samples. Though the forementioned existing methods are applicable to FFPE RNA-seq data, none of them were specifically designed and validated on FFPE samples. The mRNA expression measured from FFPE can have lower quality and higher sparsity (i.e., many zero counts occur), compared with FF samples. Consequently, the zero-inflation makes the assumption of model-based FF RNA-seq normalization invalid. For most scaling factor-based RNA-seq normalization, practitioners need to discard genes with many zeros beforehand, which may be a significant portion of the data when applied to FFPE samples. To the best of our knowledge, MIXnorm (Yin et al., 2020) is the only method that is designed to normalize FFPE RNA-seq data but that is applicable to FF RNA-seq data as well. MIXnorm addresses the zero inflation by separately modeling the expressed genes and non-expressed genes in a mixture model. Yin et al. (2020) considered normalized expression from paired FF (or like) samples as a surrogate of the truth and showed quantitatively that MIXnorm consistently outperforms commonly used FF RNA-seq normalization methods when applied on FFPE samples by comparing the gene-wise correlations. In addition, it has been shown that after MIXnorm normalization, RNA-seq data from FFPE tissues enable us to detect important up- or down-regulated genes. However, MIXnorm relies on a complex latent variable structure for model fitting, which causes a heavy computational burden for large data sets. We show in this paper that the statistical model of MIXnorm can be properly simplified to still capture the main characteristics of the FFPE RNA-seq data. We propose a simplified version of MIXnorm, labeled SMIXnorm, for FFPE RNA-seq data normalization. The fitting of SMIXnorm requires a less complicated latent variable structure. We show through simulation studies and real data applications that SMIXnorm retains almost the same performance as MIXnorm, while greatly reducing the computing time.

More importantly, there is a lack of platforms that integrate existing methods and produce normalized data by different methods. Evans et al. (2018) mentioned that the selection of normalization methods played an important role in downstream analysis due to the different assumptions those methods made. Furthermore, it is important to raise the awareness of the separate normalization methods for FFPE samples, as more and more applications involve RNA-seq data from such samples. We developed a web portal, *RNA-seq Normalization (RSeqNorm)* (http://lce.biohpc.swmed.edu/rseqnorm/), to conduct normalization for both FF and FFPE RNA-seq data. It offers seven normalization methods, with accompanying diagnostic plots for users to visually examine the RNA-seq data quality. Based on this platform, we compared different normalization methods using both comprehensive simulation studies and real data applications. These results, together with the *RSeqNorm* web portal, will facilitate users to select the best normalization method for their application.

The paper is structured as follows. In section 2.1, we present the SMIXnorm method. The statistical model of SMIXnorm is simplified from that of MIXnorm. An efficient nested Expectation-Maximization (EM) algorithm is designed for model fitting. We further justify the simplifications by comparing SMIXnorm to MIXnorm from a technical point of view. An introduction of the web portal *RSeqNorm* is given in section 2.2. Section 3 reports simulation studies and real data analyses. Finally, a brief concluding discussion is made in section 4.

## 2. Materials and Methods

### 2.1. The SMIXnorm Method

#### 2.1.1. The Simplified Statistical Model

Assume the RNA-seq count data from FFPE samples can be summarized by a matrix ***C***_*I*× *J*_, where *C*_*ij*_ is the number of reads in sample *i* for gene *j*. We adopt a similar latent variable framework as in MIXnorm to address the zero inflation of such data. That is, the binary latent variable *D*_*j*_ = 1 indicates gene *j* is expressed and *D*_*j*_ = 0 indicates gene *j* is not expressed in the study for *j* = 1, ..., *J*. Then we model the count data as a mixture of zero-inflated Poisson (ZIP) and normal distributions,

(1) $$\begin{array}{r} C_{ij}\sim \text{ZIP}\left(\pi_{j},\delta \right),\text{if}D_{j}=0, \end{array}$$

(2) $$\begin{array}{r} L_{ij}\sim \text{N}\left(\mu_{i},\sigma_{i}^{2}\right),\text{if}D_{j}=1, \end{array}$$

(3) $$\begin{array}{r} D_{j}\sim \text{Ber}\left(\phi \right), \end{array}$$

where *L*_*ij*_ = log(*C*_*ij*_+1) denotes the log transformed count, 0 ≤ π_*j*_, ϕ ≤ 1, δ, μ_*i*_, σ_*i*_≥0 for *i* = 1, ..., *I* and *j* = 1, ...*J*. The model assumes an unobserved variable *D*_*j*_ which follows a Bernoulli distribution with parameter ϕ. Genes with *D*_*j*_ = 0 are considered not expressed. These include low-expression genes that have abundance below detection limit, or biologically non-expressed genes that are absent from the biological sample of interest, or genes that should have been expressed but suffer from high-level mRNA degradation. The observed counts from non-expressed genes are due to background noise and are modeled by a zero-inflated Poisson distribution with gene-specific probability of extra zeros π_*j*_ and a common expected Poisson count δ. Genes with *D*_*j*_ = 1 are expressed genes and we model the log counts of those genes by a normal distribution with sample-specific location and scale parameters. Compared to the model of MIXnorm, we use the normal distribution in (2) instead of a truncated normal distribution and a common Poisson mean δ rather than the sample-specific mean δ_*i*_. As will be discussed in section 2.1.3, these would greatly facilitate the computation while not hurting much the performance. Note that the normal distribution assigns positive densities to negative log counts *L*_*ij*_, which never occur in real data. However, it is reasonable to assume that the negative values only take a negligible portion of the density in modeling expressed genes as the mean (log) counts of such genes are usually well above zero. Further, SMIXnorm is directly applicable to FF or like samples based on the same argument in Yin et al. (2020) that FF samples may be considered a reduced case of FFPE samples.

#### 2.1.2. Model Fitting

Let Θ = (**μ**, **σ**, **π**, δ, ϕ) denote the set of all parameters in the simplified model. The observed data likelihood as a function of Θ is defined as follow:

(4) $$\begin{array}{l} L(\Theta |C)=\prod_{j=1}^{J}p(C_{j}|\Theta )\\ \;=\prod_{j=1}^{J}[p(C_{j}|D_{j}=1,\mu ,\sigma )p(D_{j}=1|\phi )\\ \;+p(C_{j}|D_{j}=0,\pi_{j},\delta )p(D_{j}=0|\phi )]\\ \;=\prod_{j=1}^{J}[\prod_{i=1}^{i}p(C_{ij}|D_{j}=1,\mu_{i},\sigma_{i})\;\cdot \;\phi \\ \;+\prod_{i=1}^{I}p(C_{ij}|D_{j}=0,\pi_{j},\delta )\cdot (1-\phi )], \end{array}$$

where *p*(*C*_*ij*_|*D*_*j*_ = 1, μ_*i*_, σ_*i*_) is the probability density function (pdf) of *C*_*ij*_ for expressed genes such that log(*C*_*ij*_+1) follows the normal distribution in (2), and *p*(*C*_*ij*_|*D*_*j*_ = 0, π_*j*_, δ) is the zero-inflated Poisson probability mass function (pmf) for non-expressed genes as described in (1). Note that a continuous distribution is used to approximately model the discrete random variable log(*C*_*ij*_+1). See Yin et al. (2020) for a detailed justification for the approximation.

A common approach to obtain the maximum likelihood estimate (MLE) of Θ is to treat (**C**, **D**) as the complete data and update the parameter estimates iteratively by an EM algorithm. However, direct implementation of the EM algorithm requires Newton-Raphson type maximization to update the parameters within the ZIP component, which may cause the EM algorithm fail to converge due to numerical instability. By a similar approach in Yin et al. (2020), we update the parameter estimates from the ZIP component by nesting another EM algorithm and avoid the Newton-Raphson type optimization. Specifically, we construct another latent variable *Z*_*ij*_ for genes not expressed so that the ZIP distribution can be treated as a mixture of two states, the perfect zero state and the Poisson state. Assume *Z*_*ij*_ = 1 when *C*_*ij*_ is from the perfect zero state and *Z*_*ij*_ = 0 when *C*_*ij*_ is from the Poisson state, satisfying *Z*_*ij*_|*D*_*j*_ = 0 ~ Ber(π_*j*_). Let ***Y***_com_ = (***C***, ***D***, ***Z***) denotes the complete data. The complete-data log-likelihood with latent variables ***D*** and **Z** is given by

(5) $$\begin{array}{l} \ell (\Theta |C,D,Z)=\sum_{j=1}^{J}\sum_{i=1}^{I}\{D_{j}\left[\log\phi +\log\text{N}\left({\text{L}}_{\text{ij}}\text{|}\mu_{\text{i}},\sigma_{\text{i}}\right)-\log(C_{ij}+1)\right]\\ \;+(1-D_{j})[\log\left(1-\phi \right)+Z_{ij}\log\pi_{j}\\ \;+\left(1-Z_{ij}\right)\log\left(1-\pi_{j}\right)]\\ \;+\left(1-D_{j}\right)\left(1-Z_{ij}\right)\left[C_{ij}\log\delta -\delta -\log C_{ij}!\right]\}. \end{array}$$

The outer EM treats **C** as observed data and **D** as missing data. Let Θ^(*t*)^ = (**μ**^(*t*)^, **σ**^(*t*)^, **π**^(*t*)^, δ^(*t*)^, ϕ^(*t*)^) denote the set of current parameter estimates after *t* iterations of the algorithm. The distribution of ***D*** given the observed data and Θ^(*t*)^ is

(6) $$\begin{array}{r} p\left(D|C,\Theta^{\left(t\right)}\right)=\overset{J}{\underset{j=1}{\prod }}\frac{p\left(C_{j},D_{j}|\Theta^{\left(t\right)}\right)}{p\left(C_{j}|\Theta^{\left(t\right)}\right)}=\overset{J}{\underset{j=1}{\prod }}{\left(w_{j}^{\left(t\right)}\right)}^{D_{j}}{\left(1-w_{j}^{\left(t\right)}\right)}^{1-D_{j}}, \end{array}$$

where

(7) $$\begin{array}{r} w_{j}^{\left(t\right)}=\frac{\phi^{\left(t\right)}p\left(C_{j}|D_{j}=1,\mu^{\left(t\right)},\sigma^{\left(t\right)}\right)}{\phi^{\left(t\right)}p\left(C_{j}|D_{j}=1,\mu^{\left(t\right)},\sigma^{\left(t\right)}\right)+\left(1-\phi^{\left(t\right)}\right)p\left(C_{j}|D_{j}=0,\pi_{j}^{\left(t\right)},\delta^{\left(t\right)}\right)}. \end{array}$$

Note that in (5), the complete-data log-likelihood is linear in ***D***. Therefore, the outer E step, which calculates the conditional expectation of the complete-data log-likelihood with respect to the missing data **D**, is reduced to computing $w_{j}^{\left(t\right)}=\text{E}\left(D_{j}|C,\Theta^{\left(t\right)}\right)$ only once per iteration. Within each iteration of the outer EM, the inner EM repeats *K* cycles and treats **Z** as missing data and (***C***, ***w***^(*t*)^) as observed data, where $w^{\left(t\right)}=\left(w_{1}^{\left(t\right)},...,w_{J}^{\left(t\right)}\right)$. By the similar argument and that $\overset{I}{\underset{i=1}{\sum }}Z_{ij}$ is the complete-data sufficient statistic for π_*j*_, the *k*th cycle of the inner E step involving ***Z*** is reduced to calculating the conditional expectation of *Z*_*ij*_ given $\left(C,w^{\left(t\right)},\Theta^{\left(t+\frac{k-1}{K}\right)}\right)$.

Details of the nested EM algorithm are summarized in section 1 in Supplementary Material. The convergence can be detected using the change of the observed-data log-likelihood ℓ(Θ|***C***) in two consecutive iterations which is trivial to obtain via (4). We set the number of inner EM cycles *K* = 5 within each iteration in our implementation as in Yin et al. (2020).

Similar to MIXnorm, SMIXnorm normalization relies on the accurate classification of the genes that are expressed or not. This is indicated by the latent variable *D*_*j*_. We estimate *D*_*j*_ by its conditional expectation given the observed data at the last E-step. Assume that the majority of genes are not differentially expressed across samples (Robinson and Oshlack, 2010). Then the means of the normal distributions μ_*i*_'s can be treated as the sample-specific noises. The normalized expression for sample *i* and gene *j* can be given by

(8) $$\begin{array}{r} N_{ij}=\text{E}\left(D_{j}|C_{j},\hat{\Theta }\right)\times \left(L_{ij}-{\hat{\mu }}_{i}\right), \end{array}$$

where $\hat{\Theta }$ denotes the MLE of Θ. In our numerical experiments, when gene *j* is not expressed, it is often the case that the conditional expectation $\text{E}\left(D_{j}|C_{j},\hat{\Theta }\right)\approx 0$, which makes *N*_*ij*_ ≈ 0; when gene *j* is expressed, $\text{E}\left(D_{j}|C_{j},\hat{\Theta }\right)$ is often close to 1. Thus, we may simply output zero for genes with ${\hat{D}}_{j}=0$, and $L_{ij}-{\hat{\mu }}_{i}$ for genes with ${\hat{D}}_{j}=1$ in the actual implementation. The proposed method normalizes the data by subtracting the estimated sample-specific noise from the log count. Therefore, the normalized data are in the log scale.

#### 2.1.3. SMIXnorm vs. MIXnorm

There are two major simplifications when comparing SMIXnorm to MIXnorm. First, SMIXnorm assumes a common Poisson mean δ for the non-expressed genes, where MIXnorm allows the sample-specific Poisson means δ_*i*_. Note that δ appears in the normalization step (8) through the conditional expectation of *D*_*j*_ that can be computed by Equation (7). We observe in practice that after the nested EM algorithm converges, the conditional expectation $\text{E}\left(D_{j}|C,\Theta^{\left(t\right)}\right)$ [i.e., $w_{i}^{\left(t\right)}$ in Equation 7] is not sensitive to the choice of a common or sample specific Poisson mean. With the parameters set to their MLE, this conditional expectation mainly depends on the ratio between $p\left(C_{j}|D_{j}=0,{\hat{\pi }}_{j},\hat{\delta }\right)$ (or $p\left(C_{j}|D_{j}=0,{\hat{\pi }}_{j},{\hat{\delta }}_{i}\right)$ in MIXnorm) and $p\left(C_{j}|D_{j}=1,\hat{\mu },\hat{\sigma }\right)$. The Poisson mean essentially reflects the background noise level and is supposed to be a small positive number. Thus, the former distribution, regardless of using $\hat{\delta }$ or ${\hat{\delta }}_{i}$, puts most of its probability mass near 0, whereas the latter distribution in practice has negligible density around small values near 0. Consequently, the conditional expectation in Equation (7) is close to either 1 or 0 depending on whether the gene is expressed or not and reducing δ_*i*_ to δ has little effect in the normalization step.

We further simplify the model by ignoring the truncation on the normal distributions. Compared to MIXnorm, the sample-specific noise among expressed genes is captured by the mean of a normal distribution in SMIXnorm instead of the mean of a truncated normal distribution. However, we argue that these two estimates are asymptotically identical. Assume we model a set of continuous positive real numbers ***X*** = (*X*_1_, ..., *X*_*J*_) by a truncated normal distribution TN(θ, τ^2^, 0, +∞), where θ and τ are the mean and standard deviation of the corresponding normal distribution before truncation. To estimate the mean of the truncated normal distribution, which is a function of θ and τ, say *m*(θ, τ), one common approach is to first obtain the maximum likelihood estimates $\left(\hat{\theta },\hat{\tau }\right)$, then the maximum likelihood estimate of the mean is $m\left(\hat{\theta },\hat{\tau }\right)$ based on the invariance property of MLE. Another approach for a point estimate is the method of moments. The method of moments estimate of *m*(θ, τ) is simply the sample mean $\bar{X}$, which is essentially the MLE of μ if we ignore the truncation and model the data with N(μ, σ^2^). Under the large sample situation, as is often the case in RNA-seq data involving many genes from whole-genome experiments, both $m\left(\hat{\theta },\hat{\tau }\right)$ and $\bar{X}$ are asymptotically consistent estimators for *m*(θ, τ). The use of a normal distribution without truncation is appealing since it has closed-form parameter updates within the nested EM algorithm, while a truncated normal distribution requires additional latent variable structures and data augmentation to obtain closed-form parameter updates (Yin et al., 2020). Overall, SMIXnorm produces similar normalized expression compared to MIXnorm whereas greatly simplifies the model fitting process, as will be confirmed in section 3 by numerical evidence.

### 2.2. *RSeqNorm* Web Portal

**Figure 1:**
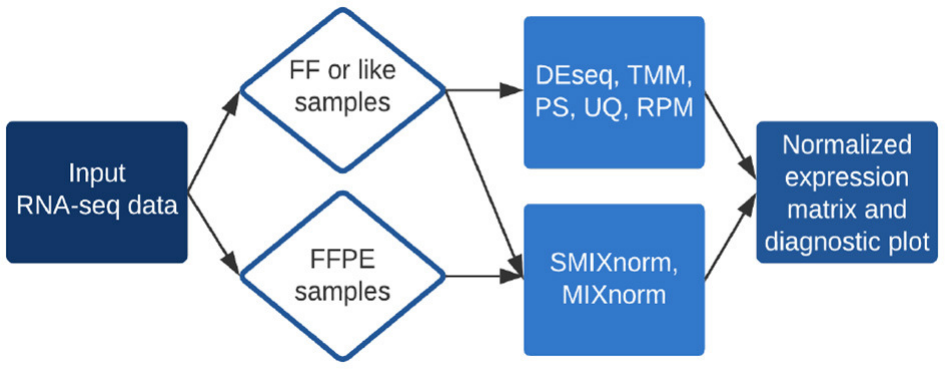
Summary of *RSeqNorm* web-portal process.

The *RSeqNorm* web portal (http://lce.biohpc.swmed.edu/rseqnorm) provides a set of analysis routines for normalization of RNA-seq data from either FF or FFPE samples. The workflow is illustrated in Figure 1. Users provide raw sequence read count data (e.g., from RNA-seq experiments) in the form of an integer-valued matrix **C**. The web portal can compute the normalized expression as well as diagnostic information for download. We implement SMIXnorm, MIXnorm and five commonly used normalization methods, including Reads Per Million (RPM), Upper-Quartile (UQ), DESeq, Trimmed Mean of *M*-values (TMM) and PoissonSeq (PS). Though developed for FFPE data, SMIXnorm and MIXnorm are directly applicable to FF data normalization. For FF data normalization, there seems to have no unanimously best normalization method. We suggest using the methods offered by *RSeqNorm* and evaluating the normalization performance using prior information or known biological knowledge. For example, users may conduct a differential expression (DE) test following the normalization step and select the method that detects more genes that are known to be differentially expressed in the literature.

**Figure 2:**
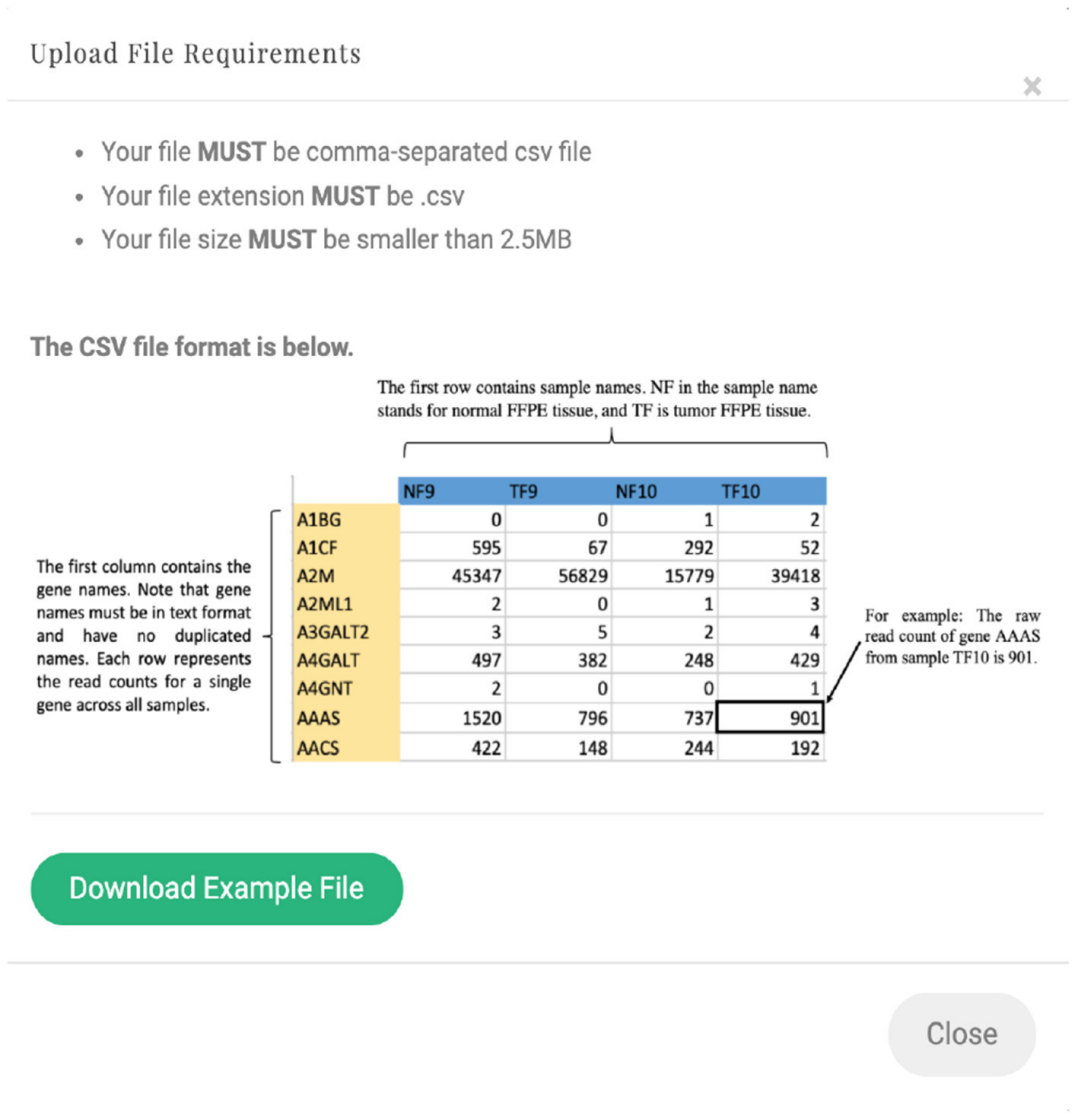
*RSeqNorm* upload file requirements.

*RSeqNorm* accepts the raw read count matrix in a comma-separated values (CSV) file. The (*i, j*) element of the count matrix records how many reads have been assigned to gene *j* in sample *i*. An example input file is downloadable from the *RSeqNorm* website. Detailed file requirements are shown in Figure 2. SMIXnorm and MIXnorm require additional input arguments including the maximum number of iterations [range (10, 50), default value 15] and the convergence threshold [recommend range (1e−5, 1), default value 0.01] for the nested EM algorithm. We note that the observed-data log-likelihood as a function of all parameters may have a large curvature near the MLE. However, the SMIXnorm and MIXnorm normalized expression values are not sensitive to small variations of the parameter estimates. Therefore, the convergence criterion here is defined as the maximum absolute change in the parameter updates between the previous and current iterations, instead of using the change in the observed-data log-likelihood. The algorithm stops when the absolute change is smaller than the predetermined threshold value or the maximum number of iterations is reached. We mentioned in section 2.1.3 that the conditional probability of being expressed is often close to either 0 or 1. As a consequence, the normalized expression level for a non-expressed gene is usually a trivial number (<10e−10 in our implementation to real data). Therefore, an approximate set of normalized expression is also available for SMIXnorm and MIXnorm, which normalizes genes with ${\hat{D}}_{j}=0$ directly to 0 across all samples, where ${\hat{D}}_{j}=I\left(w_{j}^{\left(t\right)}>c_{w}\right)$ at convergence, *I*(·) is the indicator function and *c*_*w*_ is set to 0.5 in *RSeqNorm*. In practice, we observe that the choice of *c*_*w*_ is not sensitive in identifying expressed genes. The conditional probability of being expressed ($w_{j}^{\left(t\right)}$) and the proportion of expressed genes identified ($\hat{\phi }$) are returned in the R package *RSEQNORM*, which is downloadable from our website. Note that $\hat{\phi }$ may reflect the overall data quality for an RNA-seq experiment (i.e., a value close to 1 indicates high quality).

**Figure 3:**
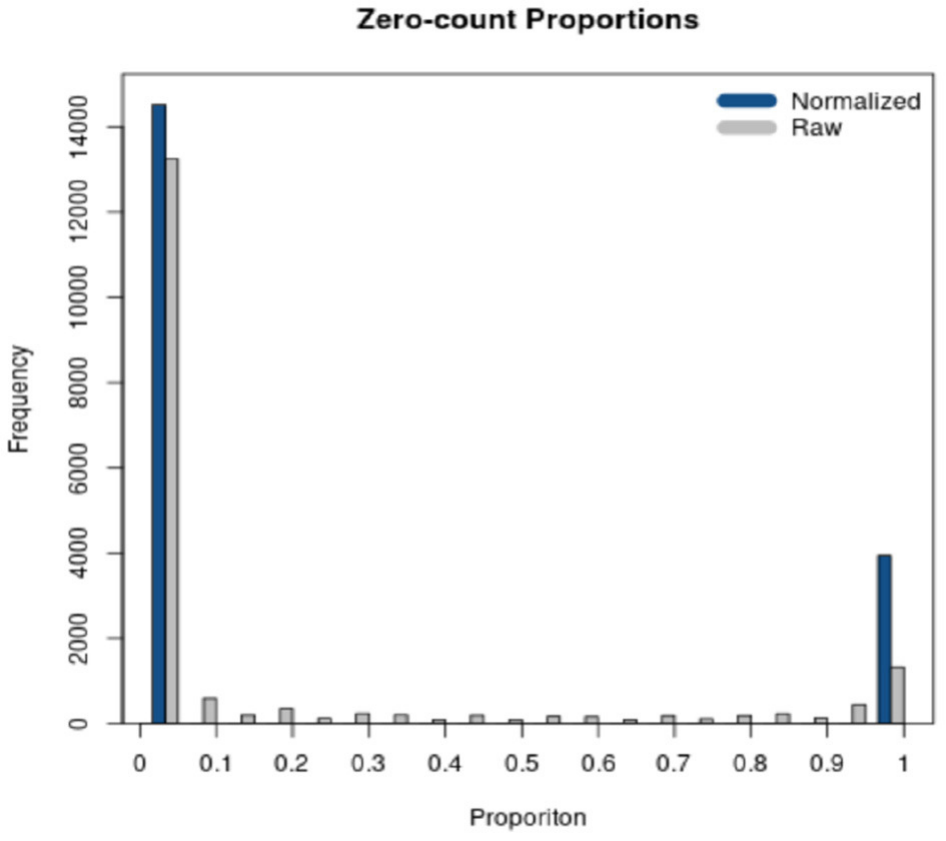
Diagnostic plot returned by *RSeqNorm* using SMIXnorm.

*RSeqNorm* returns the normalized expression in a CSV file with the same dimension as the user input file. User may leave the web portal after successfully submitting the job and an email notification will be sent to the user with a download link when the normalization is finished. A histogram of zero-count proportions is also returned as shown in Figure 3, where SMIXnorm is used on the example data provided by *RSeqNorm*. The histogram shows the distribution of zero-count proportions among all samples (represented by the horizontal axis) over all input genes. High frequencies near 0 indicate that most biologically expressed genes are actually expressed in all samples in the experiment and so the data are of high quality. Ideally, one would expect that a gene is either expressed among all samples or not expressed in any of the samples. In this ideal case, the histogram shows frequencies only at 0 and 1 on the horizontal axis.

## 3. Results

### 3.1. Simulation

We conducted simulation study to show that SMIXnorm greatly reduces computing time and maintains performance comparable to MIXnorm that is better than all FF RNA-seq normalization methods. Our simulation study evaluates the computing time of SMIXnorm and MIXnorm with different sample sizes. Note that the SMIXnorm model cannot be used to simulate the data since it permits negative log transformed counts that do not exist in practice. Here, a modified MIXnorm model was used to generate synthetic data sets (under the same settings as in Simulation VI in Yin et al., 2020). The model parameters were set to their MLEs estimated from a public RNA-seq dataset for FFPE soft tissue sarcomas samples (Lesluyes et al., 2016), which contains expression levels for 20, 242 protein-coding genes from 41 patients.

**Figure 4:**
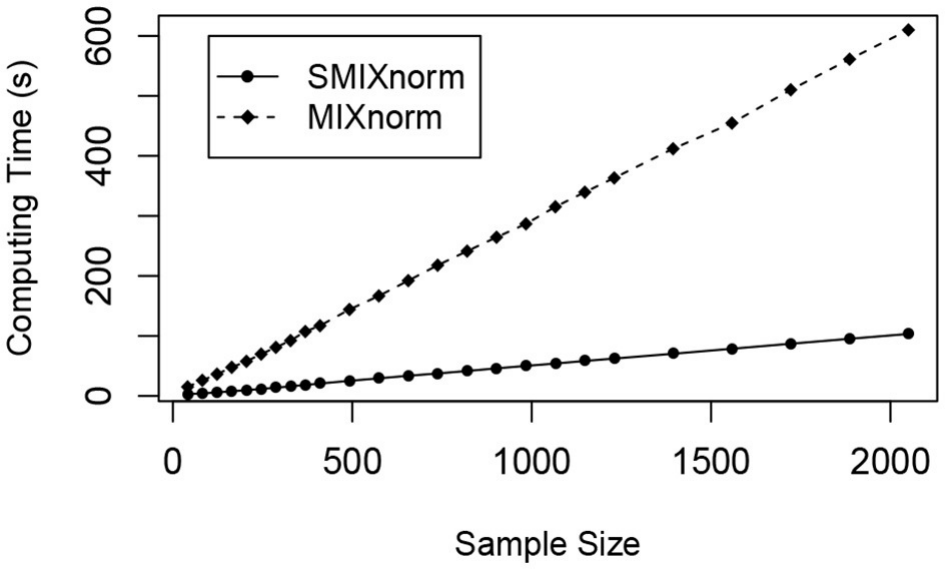
Simulation study. Average computing time of SMIXnorm and MIXnorm vs. sample size.

The sample size was set to a multiple of 41 from 41 to 2, 050. The average computing time for SMIXnorm and MIXnorm over 50 replicates is plotted against sample size in Figure 4. Both SMIXnorm and MIXnorm show a linear relationship between the average computing time and the sample size with Pearson correlations greater than 0.99. The linear regression fit of the SMIXnorm computing time against sample size results in a slope estimate of 0.051, compared to 0.297 from MIXnorm. To evaluate the performance of SMIXnorm and MIXnorm together with five commonly used FF RNA-seq normalization methods (PS, UQ, DESeq, RPM, and TMM), we calculated the gene-wise Pearson correlations for the 20, 242 genes between the normalized and true expression for each of the 50 simulated data set under the setting of 41 samples. The results are reported in Supplementary Figure 1, showing that SMIXnorm and MIXnorm have almost identical performance and they consistently beat the other methods.

### 3.2. Real Data Analysis

When dealing with real data where true expression is unknown, researchers often consider frozen sections as the gold standard for most molecular assays. As mentioned in the introduction, the frozen process maintains RNA well, compared to the FFPE process. Therefore, we used the paired FF RNA-seq expression after normalization as a surrogate to the true gene expression in our three data examples, in order to evaluate the performance of different normalization methods.

#### 3.2.1. Colorectal Cancer Data

The colorectal cancer data (Omolo et al., 2016) contains 54 selected FFPE tumor specimens from a larger multi-center cohort with paired FF samples. Gene expression levels were measured by whole genome RNA-seq (RNA-Acc) assay and Affymetrix GeneChip (Affy) platform on the FFPE samples and FF samples, respectively.

The activation of RAS signaling pathway is frequent in human cancer. Recent studies have shown that RAS mutations account for approximately 40% of colorectal cancers and lung cancers (Omolo et al., 2016). A number of RAS pathway activation gene expression signatures have been identified using multiple types of cancer cell lines and human FF samples. Omolo et al. (2016) evaluated an 18-gene RAS pathway signature on FFPE samples in five technology platforms. We focus on the Illumina whole genome RNA-seq of FFPE samples and the gold standard FF samples measured by Affymetrix GeneChip. The Affy_FF samples were normalized using the RMA method (Irizarry et al., 2003; Omolo et al., 2016).

To assess the performance of the translation of the gene signature from FF to FFPE samples, we considered the same metric as in Omolo et al. (2016). The RAS pathway activation score is defined as the mean normalized expression levels of the 18 RAS genes for each sample. We calculated the Spearman correlation using the 54 pairs of FF and FFPE RAS pathway activation scores for each normalization method and summarized the results in Table 1. The raw data give the lowest correlation as expected. SMIXnorm and MIXnorm give almost the same results and the highest correlations. The *p*-value based on a two-sided permutation test for the hypothesis *H*_0_:ρ = 0 is reported in the parenthesis. SMIXnorm, MIXnorm, PS, DESeq, RPM and UQ report significant correlations at the significance level of 0.05. MIXnorm takes about 42 s to normalize the colorectal cancer data, while SMIXnorm only takes about 7.5 s.

**Table 1:** Correlations ρ between normalized FF and FFPE RAS pathway activation scores.

	**SMIXnorm**	**MIXnorm**	**PS**	**DESeq**
ρ	0.343 (0.012)	0.343 (0.011)	0.324 (0.017)	0.298 (0.029)
	RPM	UQ	TMM	Raw
ρ	0.286 (0.036)	0.270 (0.049)	0.153 (0.269)	0.125 (0.366)

*p-values in the parenthesis are based on a two-sided permutation test for the hypothesis H_0_:ρ = 0*.

#### 3.2.2. Soft Tissue Sarcomas Data

The soft tissue sarcomas data (Lesluyes et al., 2016) measure the expression levels of 20, 242 protein-coding genes from 41 patients with paired FF and FFPE samples. To evaluate the normalization performance, the gene-wise Pearson correlations between normalized FFPE and FF expression levels (in the log scale) were computed and compared among the seven different methods (SMIXnorm, MIXnorm, DESeq, RPM, TMM, PS, and UQ). A gene expression signature, Complexity INdex in SARComas (CINSARC), which contains 67 genes, has been identified as an important prognostic factor in sarcomas using fresh frozen samples. Lesluyes et al. (2016) showed that CINSARC remains a potential prognostic factor using the FFPE RNA-seq data. Therefore, we evaluated the performance of different normalization methods on all the 20, 242 genes as well as the 67 genes in the CINSARC gene signature.

**Figure 5:**
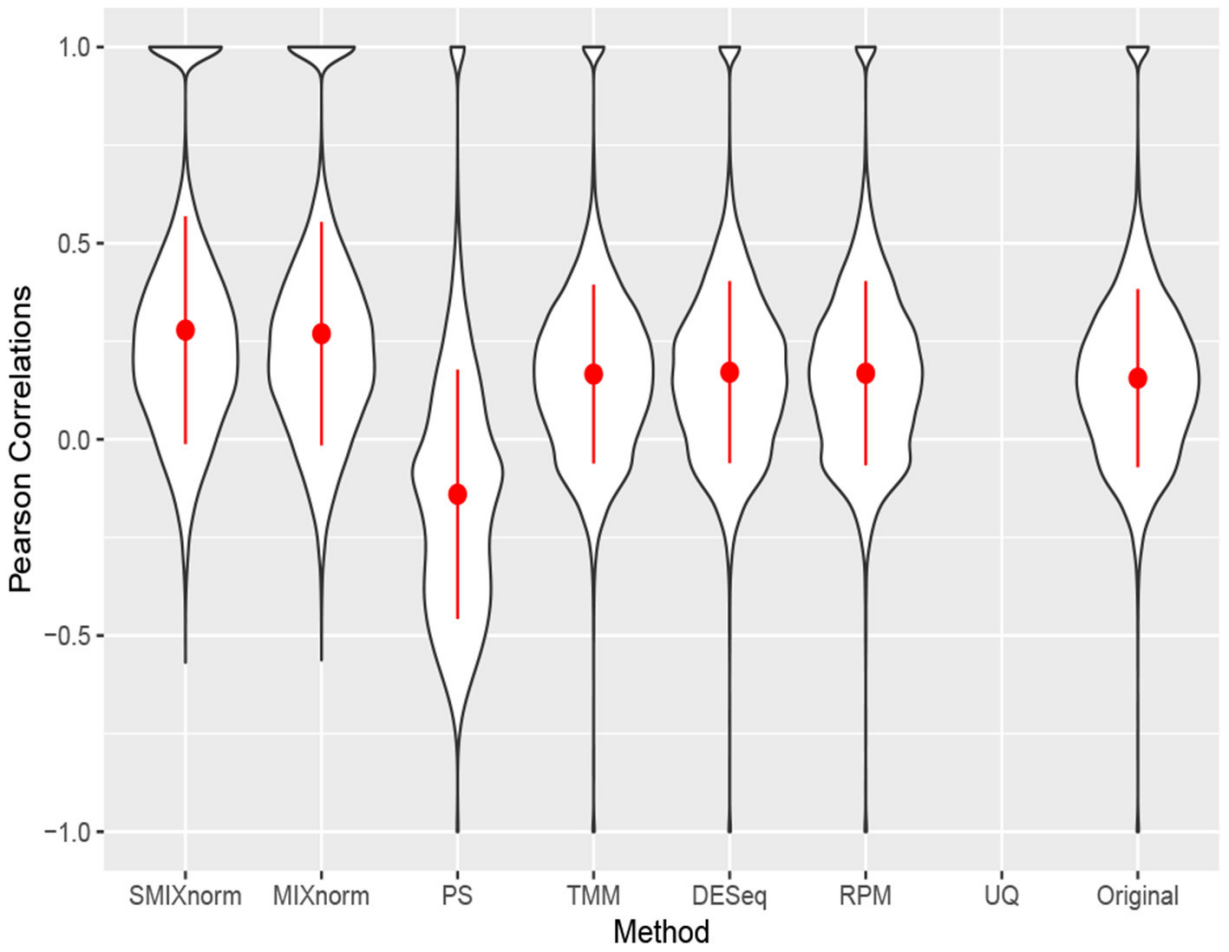
Gene-wise correlations between normalized FFPE and FF expression for soft tissue sarcomas data on all 20, 242 protein coding genes. The UQ method failed to normalize the data due to excess zero counts.

Figure 5 shows the gene-wise Pearson correlations for all the 20, 242 genes using violin plots. The dot and line in each violin plot represent the mean and standard deviation of the gene-wise Pearson correlations. The gene-wise correlations from the 67 genes in the CINSARC signature are summarized in Table 2 using the first, second, and third quartiles. Note that UQ failed to normalize the data as the scaling factor estimates equal 0 for some samples due to the excess zero counts. The genes in the gene signature have higher correlations than those calculated from the population of all protein-coding genes for all the normalization methods. The shapes of the violin plots suggest that SMIXnorm and MIXnorm have almost identical results and SMIXnorm gives the highest mean correlation while PS gives the lowest among all the methods. The three commonly used FF RNA-seq normalization methods, TMM, DESeq, and RPM, give similar results on all protein-coding genes and there is no clear improvement compared to the original correlations calculated without any normalization. However, DESeq and RPM show much higher correlations on the CINSARC gene signature compared to TMM. Overall, SMIXnorm and MIXnorm show similar results and are consistently better than the other methods. We further note that in this example, a poor choice of normalization method (e.g., UQ, TMM, or PS) may yield results worse than those from original unnormalized data. MIXnorm takes about 5 min to normalize the soft tissue sarcomas data and we note that the algorithm reaches the default maximum number of iterations before convergence. On the other hand, SMIXnorm converges in 6 iterations and takes about 10 s to normalize the data.

**Table 2:** Gene-wise correlations between normalized FFPE and FF expression for soft tissue sarcomas data on the CINSARC gene signature.

	**First Qu**.	**Median**	**Third Qu**.
SMIXnorm	0.344	0.465	0.529
MIXnorm	0.333	0.455	0.517
DESeq	0.165	0.260	0.354
RPM	0.146	0.243	0.350
TMM	0.010	0.098	0.161
PS	−0.126	0.002	0.154
UQ	–	–	–
Original	0.020	0.107	0.181

*The UQ method failed to normalize the data due to excess zero counts*.

#### 3.2.3. Clear Cell Renal Cell Carcinoma Data

**Figure 6:**
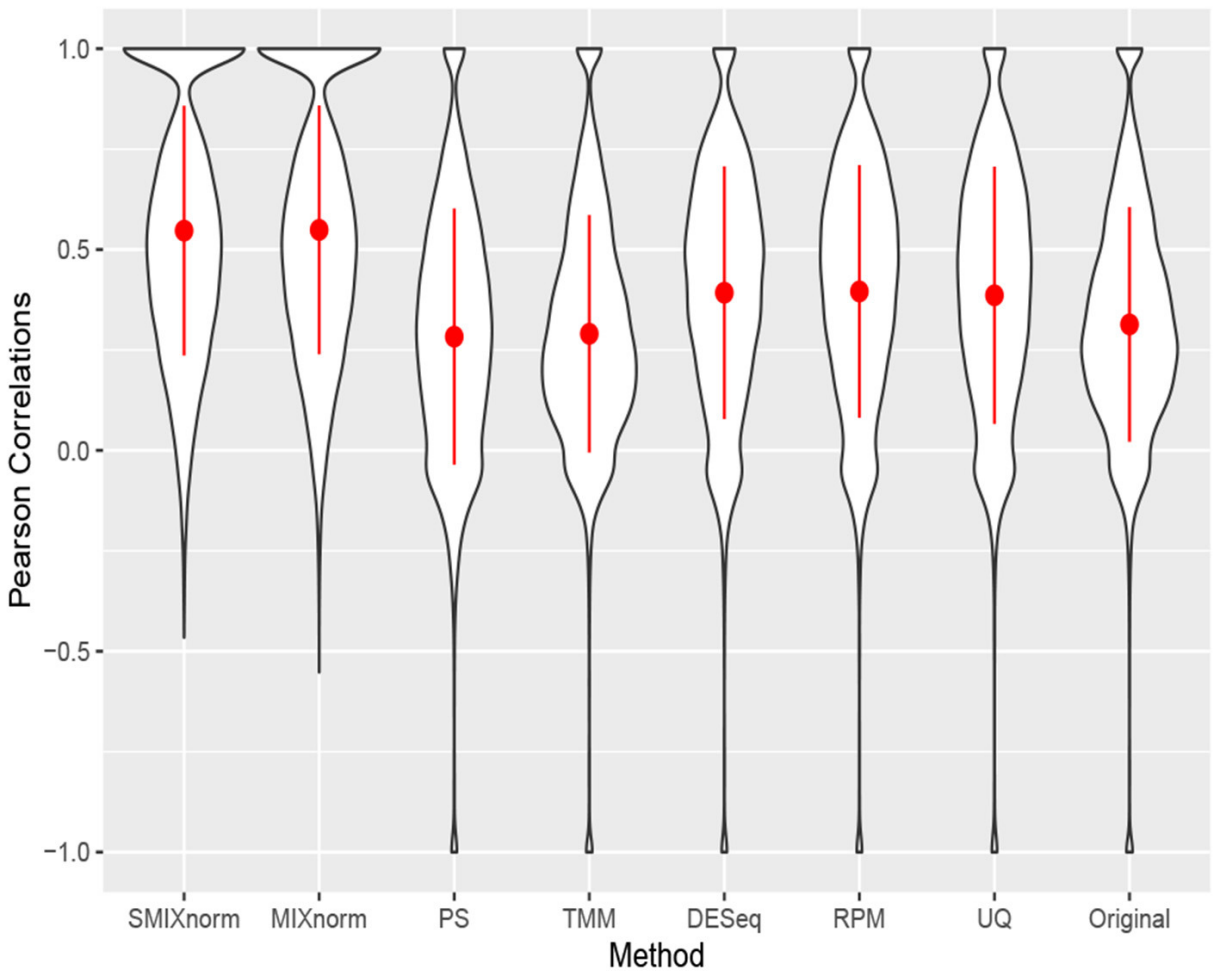
Gene-wise correlations between normalized FFPE and RNAlater for ccRCC data on 18, 458 protein coding genes.

The clear cell renal cell carcinoma (ccRCC) data are available in the repository Gene Expression Omnibus from a published study (Eikrem et al., 2016). ccRCC is the most common and aggressive histological type among the primary renal neoplasms. Metastasis is a major cause of ccRCC patient death due to the resistance to standard chemotherapy and radiotherapy. Hence, much effort has been made to unravel the underlying molecular mechanisms of ccRCC, for example, by applying gene expression analysis to develop molecular signatures of disease progression, which plays an important role in assessing the carcinogenesis and development of disease as well as guiding clinical decisions. The ccRCC RNA-seq data contain 32 pairs of FFPE and RNAlater samples with 18, 458 protein-coding genes converted from 64, 253 Ensembl annotated genes. Following Yin et al. (2020), the RNAlater samples were considered the gold standard in this study and gene-wise Pearson correlations were computed to compare the performance of the seven normalization methods. The results are shown via violin plots in Figure 6. Again, we observe that SMIXnorm and MIXnorm give almost the same results and consistently perform the best among all methods. It is interesting to note that TMM, which performs the best among existing FF normalization methods in soft tissue sarcomas data on all protein-coding genes, gives worse results than DESeq, RPM, and UQ in this application. In fact, TMM and PS show no advantage compared to the original correlations without any normalization. MIXnorm takes about 10.5 s to normalize the ccRCC FFPE data, while SMIXnorm only takes about 3.3 s.

The paired design of the ccRCC study allows us to conduct differential expression (DE) analysis between ccRCC and normal tissues. Specifically, two ccRCC and two adjacent normal tissues were obtained from each of the 16 patient. The two pairs were then stored in FFPE and RNAlater, respectively. We identified DE genes [Benjamini–Hochberg adjusted *P* < 0.05 from paired *t*-tests and absolute log2 fold change (FC) > 2] from FF and FFPE samples based on normalized expression levels and summarize results in Table 3. We find that SMIXnorm and MIXnorm give similar numbers of common DE genes from the two sources, and are much higher than the numbers from the other methods. Among the common DE genes identified from MIXnorm, SMIXnorm is able to identify 999 out of the 1, 036 genes (i.e., 96.4%). Furthermore, among the two sets of top 20 DE genes identified from RNAlater and FFPE samples, SMIXnorm and MIXnorm share the same common genes and show almost identical log2 FC (Table 4). Our analysis shows that using either SMIXnorm or MIXnorm for normalization, we are able to detect a set of up- or down-regulated genes from FFPE RNA-seq data that is similar to that from FF or like RNA-seq data.

**Table 3:** Summary of differential expression analysis based on different normalization methods from the ccRCC data.

	**FFPE**	**RNAlater**	**Common**	**Common**
	**DE genes**	**DE genes**	**DE genes**	**top 20 DE**
SMIXnorm	1,490	1,486	1,023	13
MIXnorm	1,488	1,482	1,036	13
DESeq	1,014	951	680	7
RPM	999	926	676	9
TMM	1,073	1,067	632	7
PS	1,001	1,300	652	8
UQ	1,002	943	679	8
Original	1,041	1,096	646	9

*The second column is the number of DE genes identified from the FFPE data; the third column is the number of DE genes identified from the RNAlater data; the fourth column is the number of common genes between the two sets of DE genes; the last column is the number of common genes among the two sets of top 20 DE genes from FFPE and RNAlater*.

**Table 4:** Shared DE genes among the two sets of top 20 DE genes from FFPE and RNAlater samples in the ccRCC data, ordered by the absolute value of the SMIXnorm normalized RNAlater log2 FC.

	**SMIXnorm RNAlater log2 FC**	**MIXnorm RNAlater log2 FC**	**SMIXnorm FFPE log2 FC**	**MIXnorm FFPE log2 FC**
CA9	8.02	8.04	5.66	5.66
SLC6A3	7.20	7.22	6.30	6.31
NDUFA4L2	6.38	6.39	4.88	4.89
UMOD	−6.17	−6.15	−5.63	−5.62
GP2	−5.53	−5.51	−4.96	−4.96
CLCNKA	−5.29	−5.28	−5.70	−5.69
CDCA2	5.21	5.23	5.04	5.05
TNFAIP6	5.16	5.17	5.45	5.45
SLC4A11	−5.10	−5.08	−5.23	−5.22
KNG1	−5.04	−5.02	−5.04	−5.03
SLC12A1	−4.96	−4.95	−4.90	−4.89
AQP2	−4.94	−4.92	−4.90	−4.89
NELL1	−4.79	−4.77	−5.03	−5.02

## 4. Discussions

We have developed an efficient normalization method, named SMIXnorm, for FFPE RNA-seq data normalization. Modified from the MIXnorm statistical model, we use a similar two-component mixture model to separately model the expressed and non-expressed genes. The simplifications of the statistical model avoid the complex likelihood function and the need of a complicated latent variable structure to invoke the nested EM algorithm. We have shown through real data applications and simulation studies that SMIXnorm greatly reduces the complexity of the likelihood function and the computing time without sacrificing the performance. Though other FF normalization methods take only about 1 s to normalize each dataset in our three data applications, their performance is not comparable to that of SMIXnorm and MIXnorm for FFPE samples. Some FF normalization methods perform even worse than the use of original data without any normalization.

We mentioned in the soft tissue sarcomas application that MIXnorm failed to converge at the default maximum number of iterations and tolerance level. Due to the severely different RNA degradation levels among the 41 soft tissue sarcomas FFPE samples, 10 FFPE samples have more than 10, 000 zero counts and 9 FFPE samples have less than 3, 000 zero counts. Consequently, MIXnorm gives negative estimated sample-specific location parameters of the truncated normal distributions for those samples with a higher proportion of zero counts, which blurs the distinction between expressed and non-expressed genes. SMIXnorm models the expressed genes by normal distributions without truncation, which naturally constrains the location parameters to be non-negative in all the EM iterations as sample means are used for estimation. Thus, SMIXnorm seems to be more robust and converges faster than MIXnorm.

Recently, single-cell RNA sequencing (scRNA-seq) becomes an important technology in molecular analysis. While bulk RNA-seq measures the expression in the population level across cells, scRNA-seq allows for the cell level resolution and therefore, reveals heterogeneity of cell subpopulations. Similar to FFPE RNA-seq data, a prominent feature of scRNA-seq data is the sparsity. The high proportion of zero count arises for both biological reasons and technical reasons (Lun et al., 2016; Vallejos et al., 2017). The most commonly used scRNA-seq normalization method is SCnorm (Bacher et al., 2017), which uses quantile regression to group genes by estimated count-depth relationships, and then estimates different scaling factors within each group via a second quantile regression. Other popular scRNA-seq normalization methods, including BASiCS (Vallejos et al., 2015), SAMstrt (Katayama et al., 2013), and GRM (Vallejos et al., 2017), rely on spike-ins. Therefore, most scRNA-seq normalization methods are not directly applicable to the FFPE RNA-seq data that typically do not have spike-ins.

With the rapid adaption of FFPE samples in RNA-seq analysis, it is important for users to realize that different normalization procedures should be used for FFPE vs. FF data. We offer *RSeqNorm*, a comprehensive and user-friendly normalization toolkit for RNA-seq data. To the best of our knowledge, *RSeqNorm* is the only available web-based tool that integrates different normalization methods for both FFPE and FF RNA-seq data. It includes seven normalization methods, among which five are commonly used (RPM, UQ, DESeq, TMM, and PS) for FF or like RNA-seq data. Though MIXnorm and SMIXnorm are specifically designed for FFPE RNA-seq normalization, they can be applied to FF data directly. However, it is generally inappropriate to implement other existing methods on FFPE data. The input for all methods is a read count matrix at the gene level. The output is an expression matrix after normalization with the same dimension as the input data. The R package, RSEQNORM, which implements SMIXnorm and MIXnorm is downloadable from the website (http://lce.biohpc.swmed.edu/rseqnorm).

## Data Availability Statement

The original contributions presented in the study are included in the article/Supplementary Material, further inquiries can be directed to the corresponding author/s.

## Author Contributions

SY and XW proposed and implemented the SMIXnorm method. SY, XZ, XW, and GX performed data analysis and wrote the manuscript. XZ, GX, and YX provided resources and helpful discussions. SY, XZ, and BY developed the web portal *RSeqNorm*. All authors contributed to the article and approved the submitted version.

## Conflict of Interest

The authors declare that the research was conducted in the absence of any commercial or financial relationships that could be construed as a potential conflict of interest.
