# Supplementary material for: SMIXnorm: Fast and Accurate RNA-Seq Data Normalization for Formalin-Fixed Paraffin-Embedded Samples
Source: Front Genet. 2021 Mar 24;12:650795. doi: 10.3389/fgene.2021.650795 (PMC8024626; doi:10.3389/fgene.2021.650795)
Supplement: Supplementary file 1 [file Data_Sheet_1.PDF]

frontiers

# *Supplementary Material for SMIXnorm: Fast and Accurate RNA-seq Data Normalization for Formalin-Fixed Paraffin-Embedded Samples*

## 1 NESTED EM ALGORITHM FOR SMIXNORM

Consider the raw RNA-seq count data summarized in a matrix $\boldsymbol{C}$, where the $(i, j)$ element $C_{ij}$ denotes the number of reads measured for sample $i$ gene $j$ and $L_{ij} = \log(C_{ij} + 1)$ for $i = 1, ..., I$ and $j = 1, .., J$. Suppose we knew, through a binary latent variable $D_j$, if gene $j$ is truly expressed or not. Then we model the count data as a mixture of zero-inflated Poisson (ZIP) and normal distribution,

$$C_{ij} \sim \text{ZIP}(\pi_j, \delta), \text{if } D_j = 0, \tag{S1}$$

$$L_{ij} \sim \text{N}\ (\mu_i, \sigma_i^2), \text{if } D_j = 1, \tag{S2}$$

$$D_j \sim \text{Ber}(\phi), \tag{S3}$$

To obtain the maximum likelihood estimates (MLE), direct implementation of the EM algorithm requires Newton-Raphson type optimization for parameters from the ZIP component, which may suffer from both numerical stability and computational efficiency issues (van Dyk 2000; Yin *et al.* 2020). Note that ZIP distribution can be thought of as a mixture of the perfect zero state and the Poisson state. For each non-expressed gene $j$, we introduce another binary latent variable $Z_{ij}$. Assume $C_{ij}$ is from the perfect zero state if $Z_{ij} = 1$ and $C_{ij}$ is from the Poisson state if $Z_{ij} = 0$. Obviously, $Z_{ij}|D_j = 0 \sim \text{Ber}(\pi_j)$. The complete data log-likelihood with latent variables $\boldsymbol{D}$ and $\mathbf{Z}$ is given by

$$\begin{aligned}\ell(\Theta|\mathbf{C}, \boldsymbol{D}, \mathbf{Z}) = \sum_{j=1}^{J}\sum_{i=1}^{I}\Big\{ & D_j\left[\log\phi + \log \text{N}(L_{ij}|\mu_i, \sigma_i) - \log(C_{ij}+1)\right] \\ & + (1-D_j)\left[\log(1-\phi) + Z_{ij}\log\pi_j + (1-Z_{ij})\log(1-\pi_j)\right] \\ & + (1-D_j)(1-Z_{ij})\left[C_{ij}\log\delta - \delta - \log C_{ij}!\right]\Big\},\end{aligned} \tag{S4}$$

where $\Theta$ denote the set of all parameters. Let $t$ be the current iteration of the nested EM algorithm. Following van Dyk (2000) and Yin *et al.* (2020), the nested EM algorithm first treats $\boldsymbol{C}$ as observed data and $\boldsymbol{D}$ as missing data in the outer EM and calculates the conditional expectation $w_j^{(t+1)} = \mathbf{E}\left(D_j|\boldsymbol{C}_j, \Theta^{(t)}\right)$ by

$$w_j^{(t+1)} = \frac{\phi^{(t)}p(\boldsymbol{C}_j|D_j = 1, \boldsymbol{\mu}^{(t)}, \boldsymbol{\sigma}^{(t)})}{\phi^{(t)}p(\boldsymbol{C}_j|D_j = 1, \boldsymbol{\mu}^{(t)}, \boldsymbol{\sigma}^{(t)}) + (1-\phi^{(t)})p(\boldsymbol{C}_j|D_j = 0, \pi_j^{(t)}, \delta^{(t)})}, \tag{S5}$$

where $p(\boldsymbol{C}_j|D_j = 1, \boldsymbol{\mu}^{(t)}, \boldsymbol{\sigma}^{(t)})$ is the normal probability density function and $p(\boldsymbol{C}_j|D_j = 0, \pi_j^{(t)}, \delta^{(t)})$ is the ZIP probability mass function as described in (S1) and (S2). The inner EM treats $(\boldsymbol{C}, \boldsymbol{w}^{(t+1)})$ as

observed data and $\boldsymbol{Z}$ as missing data and calculates $Z_{ij}^{\left(t+\frac{k-1}{K}\right)}$ by

$$\begin{aligned} Z_{ij}^{\left(t+\frac{k-1}{K}\right)} &= \mathbf{E}\left(Z_{ij}|C_{ij}, w_j^{(t+1)}, \Theta^{\left(t+\frac{k-1}{K}\right)}\right) = \Pr\left(Z_{ij}=1|C_{ij}, w_j^{(t+1)}, \Theta^{\left(t+\frac{k-1}{K}\right)}\right) \\ &= \frac{p\left(Z_{ij}=1, C_{ij}, w_j^{(t+1)}|\Theta^{\left(t+\frac{k-1}{K}\right)}\right)}{p\left(Z_{ij}=1, C_{ij}, w_j^{(t+1)}|\Theta^{\left(t+\frac{k-1}{K}\right)}\right) + p\left(Z_{ij}=0, C_{ij}, w_j^{(t+1)}|\Theta^{\left(t+\frac{k-1}{K}\right)}\right)} \\ &= \begin{cases} \dfrac{\left(\pi_j^{\left(t+\frac{k-1}{K}\right)}\right)^{1-w_j^{(t+1)}}}{\left(\pi_j^{\left(t+\frac{k-1}{K}\right)}\right)^{1-w_j^{(t+1)}} + \left(1-\pi_j^{\left(t+\frac{k-1}{K}\right)}\right)^{1-w_j^{(t+1)}} \exp\left(-\delta_i^{\left(t+\frac{k-1}{K}\right)}\right)^{1-w_j^{(t+1)}}} & \text{if } C_{ij}=0; \\ 0 & \text{if } C_{ij}\neq 0, \end{cases} \end{aligned} \tag{S6}$$

where $k = 1, ..., K$ is the current cycle of the inner EM. The maximization step maximizes the conditional expected complete data log-likelihood, which can be obtained by replacing $\boldsymbol{D}$ and $\boldsymbol{Z}$ by $\boldsymbol{w}^{(t+1)}$ and $\boldsymbol{Z}^{\left(t+\frac{k-1}{K}\right)}$ in (S4), with respect to $\Theta$. The proposed nested EM algorithm is summarized as follow:

---

**Algorithm 1** The nested EM algorithm

---

**Require:** $t = 0$, convergence criteria $\epsilon$, tolerance $= \epsilon + 1$, initialize $(\mu_i^{(0)}, \sigma_i^{(0)}, \pi_j^{(0)}, \delta^{(0)}, \phi^{(0)})$

**while** tolerance $> \epsilon$ **do**

Calculate

$w_j^{(t+1)} = \mathbf{E}\left(D_j|\boldsymbol{C}_j, \Theta^{(t)}\right)$ from (S5)

**for** $k$ in $1, 2, ..., K$ **do**

Calculate

$Z_{ij}^{\left(t+\frac{k-1}{K}\right)} = \mathbf{E}\left(Z_{ij}|C_{ij}, w_j^{(t+1)}, \Theta^{\left(t+\frac{k-1}{K}\right)}\right)$ from (S6),

Update

$$\pi_j^{\left(t+\frac{k}{K}\right)} = \frac{\sum_{i=1}^I Z_{ij}^{\left(t+\frac{k-1}{K}\right)}}{I}, \quad \delta^{\left(t+\frac{k}{K}\right)} = \frac{\sum_{j=1}^J \left(1-w_j^{(t+1)}\right)\left(1-Z_{ij}^{\left(t+\frac{k-1}{K}\right)}\right) C_{ij}}{\sum_{j=1}^J \left(1-w_j^{(t+1)}\right)\left(1-Z_{ij}^{\left(t+\frac{k-1}{K}\right)}\right)}$$

**end for**

Update

$\phi^{(t+1)} = \frac{\sum_{j=1}^J w_j^{(t+1)}}{J}$

$\mu_i^{(t+1)} = \frac{\sum_{j=1}^J w_j^{(t+1)} L_{ij}}{\sum_{j=1}^J w_j^{(t+1)}}, \quad \sigma_i^{(t+1)} = \sqrt{\frac{\sum_{j=1}^J w_j^{(t+1)} L_{ij}^2}{\sum_{j=1}^J w_j^{(t+1)}} - \left(\mu_i^{(t+1)}\right)^2}$

Set $\left(\boldsymbol{\pi}^{(t+1)}, \delta^{(t+1)}\right) = \left(\boldsymbol{\pi}^{\left(t+\frac{K}{K}\right)}, \delta^{\left(t+\frac{K}{K}\right)}\right)$

tolerance $= |[\ell(\Theta^{(t)}|\boldsymbol{C}) - \ell(\Theta^{(t+1)}|\boldsymbol{C})]/\ell(\Theta^{(t)}|\boldsymbol{C})|$

$t = t + 1$

**end while**

**return** MLE for all the parameters $\hat{\Theta}$

---

## 2 ADDITIONAL SIMULATION RESULTS

The following modified MIXnorm model (Yin *et al.* 2020) was used to generate synthetic data sets:

$$C_{ij} \sim \text{ZIP}(\pi_j, \delta_i), \text{for } j > \phi J,$$

$$L_{ij} \sim \text{TN}\left(\mu_i + g_j, \sigma_i^2, 0, \infty\right), \text{for } j \leq \phi J,$$

$$g_j \sim \text{N}(0, \sigma_g^2),$$

where $g_j$ is extra gene-wise noise and $\sigma_g = 1.5$. Other model parameters were set to their MLEs estimated from a public RNA-seq dataset from FFPE soft tissue sarcomas samples (Lesluyes *et al.* 2016), which contains expression levels for $20,242$ protein-coding genes from $41$ patients.

The performance of seven normalization methods is evaluated by the gene-wise Pearson correlations for the $20,242$ genes between the normalized and true expression. Fig. S1 summarizes the correlation quantiles for the $50$ simulated data sets under the setting of $41$ samples. SMIXnorm and MIXnorm show almost identical correlation quartiles and have significantly higher quartiles than others. TMM performs the best among the five existing FF RNA-seq normalization methods. The most straightforward method, RPM, gives even worse results than the use of original data without any normalization.

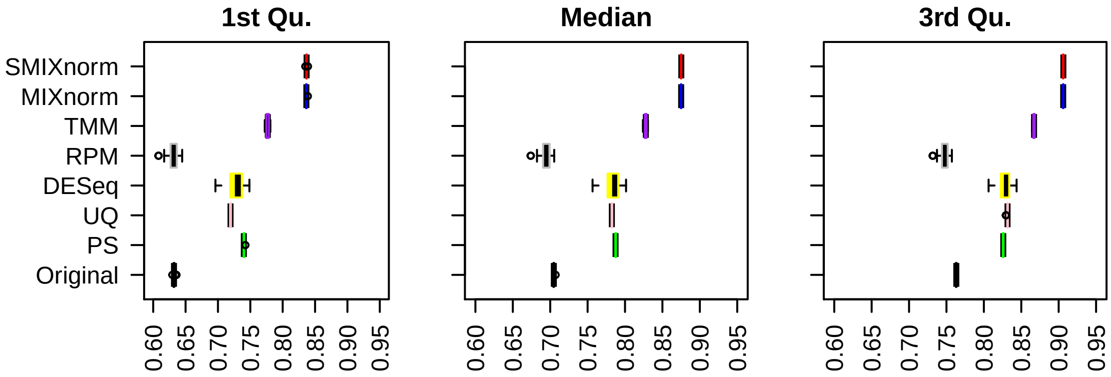


**Figure S1.** Simulation study I. The 1st to 3rd quartiles of gene-wise Pearson correlations for $20,242$ genes between the normalized and true expression for $50$ simulated data set under the setting of $41$ samples.

## REFERENCES

Lesluyes, T., Pérot, G., Largeau, M. R., Brulard, C., Lagarde, P., Dapremont, V., Lucchesi, C., Neuville, A., Terrier, P., Vince-Ranchère, D., *et al.* (2016). Rna sequencing validation of the complexity index in sarcomas prognostic signature. *European Journal of Cancer*, **57**, 104–111.

van Dyk, D. A. (2000). Nesting EM Algorithms for Computational Efficiency. *Statistica Sinica*, **10**(1), 203–225.

Yin, S., Wang, X., Jia, G., and Xie, Y. (2020). Mixnorm: normalizing rna-seq data from formalin-fixed paraffin-embedded samples. *Bioinformatics*, **36**(11), 3401–3408.
